# Altered Large-Scale Brain Functional Connectivity in Ocular Hypertension

**DOI:** 10.3389/fnins.2020.00146

**Published:** 2020-03-05

**Authors:** Antonio Giorgio, Jian Zhang, Francesco Costantino, Nicola De Stefano, Paolo Frezzotti

**Affiliations:** Department of Medicine, Surgery and Neuroscience, University of Siena, Siena, Italy

**Keywords:** glaucoma, MRI, connectivity, neurodegeneration, resting state networks, Alzheimer disease

## Abstract

We hypothesized that assessment of brain connectivity may shed light on the underpinnings of ocular hypertension (OHT), characterized by raised intraocular pressure (IOP) and no typical glaucomatous findings. OHT carries a risk for future glaucoma development, thus representing a model of presymptomatic condition. In previous studies on glaucoma, we showed altered brain connectivity since the early stage and in case of normal IOP. In this pilot study, we used a multimodal MRI approach by modeling voxelwise measures of gray matter volume, anatomical connectivity along white matter(WM) tracts, and large-scale functional connectivity in OHT subjects (*n* = 18, age: 58.3 ± 9.8 years) and demographically matched normal controls (*n* = 29). While OHT brain had no structural alterations, it showed significantly decreased functional connectivity in key cognitive networks [default mode network, frontoparietal working memory network (WMN), ventral attention network (VAN), and salience network (SN)] and altered long-range functional connectivity, which was decreased between default mode and SNs and increased between primary and secondary visual networks (VN). Overall, such findings seem to delineate a complex neuroplasticity in the OHT brain, where decreased functional connectivity in non-visual networks may reflect a type of temporarily downregulated functional reserve while increased functional connectivity between VN may be viewed as a very early attempt of adaptive functional reorganization of the visual system.

## Introduction

The term ocular hypertension (OHT) is traditionally applied to a condition with raised intraocular pressure (IOP) (≥22 mmHg or >2 standard deviations above the mean value) without typical glaucomatous visual field deficits, alterations of the optic nerve head, and retinal ganglion cell degeneration.

The prevalence estimates for OHT in the general population range from 4.5 to 9.4% for people aged >40 years, with an increasing trend with aging (Burr et al., 2012). Data from longitudinal studies indicate that development of primary open angle glaucoma (POAG) within 5 years occurs in around 10% of people with untreated OHT and in 5% of those on medication (Weinreb et al., 2014). Because of this risk, OHT may thus represent a model of presymptomatic condition.

Higher IOP is currently the only modifiable risk factor for glaucoma and is consistently associated with glaucomatous damage (Klein et al., 1992; Nemesure et al., 2007; Jiang et al., 2012). IOP reduction is still the main treatment option available (Leske et al., 2003; Chauhan et al., 2010) although only a 10–19% lower risk of progression seems to occur for every mmHg of IOP reduction (CN-TGS Group, 1998; The Agis Investigators, 2000; Chauhan et al., 2010).

This supports the theory that other factors undetectable on standard clinical examination may influence the progression of glaucoma.

Advanced magnetic resonance imaging (MRI) techniques represent a unique non-invasive approach to explore the pathogenic mechanisms of brain conditions. A number of recent studies on glaucoma have provided evidence of a neurodegenerative process across brain both within and beyond visual system (Nuzzi et al., 2018) and of various similarities with Alzheimer disease so that glaucoma may be no longer be considered a pure eye disease but rather a multifaceted and complex neurodegenerative condition.

In previous studies on glaucoma, we showed altered MRI-derived brain connectivity since the early stage (Frezzotti et al., 2016) and in case of normal IOP (Giorgio et al., 2018) and regional brain atrophy in the more advanced POAG stage (Frezzotti et al., 2014).

However, the effect of raised IOP *per se* (i.e., without evidence of visual damage) on brain structure and function has never been investigated. We hypothesized that altered brain connectivity and/or atrophy may already be present at the OHT stage as in other glaucomatous conditions, and for this reason, we performed a multimodal MRI exploratory study.

## Materials and Methods

### Study Subjects and Ophthalmological Evaluation

We recruited 18 patients with OHT (age: 58.3 ± 9.8 years, sex: 10 male) among those who were consecutively referring to the Glaucoma Service of the University of Siena. All OHT patients were on pharmacological treatment for glaucoma. Inclusion criteria for OHT patients were corneal thickness between 520 and 580 μm, absence of glaucomatous damage to the optic nerve head, glaucomatous visual field damage, and open angle at gonioscopy. Moreover, all patients underwent analysis of the optic nerve head with measurement of the retinal nerve fiber layer using optical coherence tomography (Cirrus-OCT^®^ Carl Zeiss Meditec, Dublin, CA, United States). Exclusion criteria for OHT patients were age >80 years, any ocular disorder other than OHT, any neurological disorder, use of medications potentially affecting the visual field, presence of significant cerebrovascular findings on MRI [i.e., white matter (WM) hyperintensities of grade 3 on the Fazekas scale] (Fazekas et al., 1987) or WM hyperintensities fulfilling the MRI criteria for multiple sclerosis (Polman et al., 2011) or radiologically isolated syndrome (Okuda et al., 2009).

Data from OHT subjects were compared with those of 29 normal controls (NC, age = 57.9 ± 9.9 years, 15 male), who were recruited among laboratory and hospital workers, had normal neurological and ophthalmological examinations, and had no history of neurological or ophthalmological disorders.

The study received approval from the local Ethics Committee (Azienda Ospedaliera Universitaria Senese). Informed written consent was obtained from all subjects before study entry.

### MRI Data Acquisition

In all subjects, brain MRI was acquired at the University of Siena on a 1.5-T Philips Gyroscan (Philips Medical Systems, Best, Netherlands). A sagittal survey image was used to identify the anterior and posterior commissures. Sequences were acquired in the axial plane parallel to the commissural line. A FLAIR (fluid attenuated inversion recovery) image [repetition time (TR) = 9000 ms, echo time (TE) = 150 ms, inversion recovery delay = 2725 ms, voxel size = 1 × 1 × 3 mm] was acquired for the assessment of WM hyperintensities. DTI (diffusion tensor imaging) data consisted of echo-planar imaging (EPI) (TR = 8500 ms; TE = 100 ms; voxel size = 2.5 mm^3^), with diffusion weighting distributed in 32 directions and *b*-value = 1000 s mm^–2^. Resting-functional MRI (FMRI) data were 190 volumes of EPI sequence with TR = 1000 ms, TE = 50 ms, voxel size = 3.75 × 3.75 × 6 mm. A high-resolution T1-weighted image (TR = 25 ms, TE = 4.6 ms, voxel size = 1 mm^3^) was acquired for image registration, anatomical mapping, and analysis of gray matter (GM) volume.

### MRI Data Analysis

It was performed at the Quantitative Neuroimaging Laboratory (QNL) of the University of Siena. All images were visually assessed to rule out artifacts or incidental findings.

#### WM Hyperintensities

A single observer with longstanding MRI experience (AG) performed on FLAIR images the grading of WM hyperintensities using Fazekas scale.

#### Voxelwise Analysis of FA Images

Diffusion tensor imaging data were firstly preprocessed with DTIPrep^1^ (Oguz et al., 2014), a tool for automatic quality control that minimizes various types of artifacts. Then, we used FDT (FMRIB Diffusion Toolbox), part of FSL (FMRIB Software Library^2^) (Smith et al., 2004; Jenkinson et al., 2012), to obtain, by fitting a diffusion tensor model, images of fractional anisotropy (FA), whose measure is a proxy for WM fiber integrity and thus anatomical connectivity. The subsequent analysis was performed with TBSS (Tract-Based Spatial Statistics), which allowed the registration of FA images onto a common standard space (FMRIB58_FA image) using FNIRT (FMRIB Non-linear Image Registration Tool) and to create, from the mean FA images, the mean WM “skeleton” (thresholded at FA > 0.2), representing the centers of all tracts common to the whole study population, onto which registered FA images were finally projected.

#### Voxelwise Analysis of GM Volumes

It was performed on 3D T1-weighted images with FSL-VBM (Voxel-Based Morphometry) (Douaud et al., 2007), which uses an optimized VBM protocol (Good et al., 2001), in line with a previously used procedure (Frezzotti et al., 2014, 2016; Giorgio et al., 2015). Briefly, T1-W images were brain-extracted with BET (Brain Extraction Tool), GM-segmented with FAST (FMRIB Automated Software Tool), and registered onto the MNI152 standard image using FNIRT. Then, all native GM images were non-linearly registered onto a symmetric (i.e., with the same number of subjects in each group) study-specific GM template, modulated, and smoothed (isotropic Gaussian kernel, sigma = 3 mm).

#### Voxelwise Analysis of Resting FMRI

For each subject, various preprocessing steps were performed on resting FMRI images: removal of the first five volumes to allow signal stability; initial motion correction by volume realignment to the middle volume using MCFLIRT (FMRIB’s Linear Image Registration Tool) (Jenkinson and Smith, 2001); non-brain removal using BET; global 4D mean intensity normalization; spatial smoothing [6 mm FWHM (full width at half maximum)]; registration to the T1-weighted image using affine boundary-based registration cost as implemented in FLIRT (FMRIB Linear Image Registration Tool) and subsequent transformation onto MNI152 standard space using non-linear registration FNIRT (warp resolution: 10 mm); use of ICA-AROMA [independent component analysis (ICA)-based automatic removal of motion artifacts^3^ ] in order to further remove motion-related artifacts (Pruim et al., 2015); regression of WM and cerebrospinal fluid (both thresholded at a very conservative threshold of 95% tissue probability) in order to remove residual structured noise; application of a high-pass temporal filtering (cutoff frequency: 100 s); final normalization to MNI152 standard space using FNIRT. The filtered, normalized FMRI images of all study subjects were concatenated into a single 4D image, which was then automatically decomposed by MELODIC (Multivariate Exploratory Linear Optimized Decomposition into ICs^4^) (Beckmann and Smith, 2004; Beckmann et al., 2005) into a set of 16 ICs, a number which were automatically estimated using the Laplace approximation to the Bayesian evidence of the model order. ICs of interest were selected by visual inspection and by comparison with previously described resting state networks (RSNs) (Smith et al., 2009) and reflect “coactivation” or “synchronization” across the network. Two ICs represented noise (cerebrospinal fluid pulsations and motion) and were thus discarded before further processing.

Finally, voxelwise intranetwork (short-range) functional connectivity analysis was performed using the “dual-regression” approach, where the set of spatial maps from the group-average analysis was used to generate subject-specific versions of the spatial maps and associated time series (Beckmann et al., 2009). The outputs of the first-stage dual regression, i.e., the subject-specific time series, were used for estimating temporal correlation between all the RSN pairs, which is a measure of internetwork (or long-range) functional connectivity strength, using FSLNets^5^. All internetwork Pearson *r* correlation coefficients were transformed into *z*-scores using the Fisher transform to improve data normality.

### Statistical Analysis

As for general statistics, the between-group differences were tested with *t* tests for age and with chi-square test for sex and grade of WM hyperintensities. Data were considered significant at *p* < 0.05. SPSS was used to perform such statistical analyses.

As for voxelwise analysis, group differences in FA along WM tracts (anatomical connectivity), GM volumes, and functional connectivity at the level of RSNs were performed in the general linear model framework with *t* tests using “randomize,” a non-parametric permutation testing (*n* = 5000) (Winkler et al., 2014). Following a previous approach (Frezzotti et al., 2014, 2016; Giorgio et al., 2018), thresholding of statistical images was performed with TFCE (Threshold-Free Cluster Enhancement), with a significance level of *p* ≤ 0.005, uncorrected for multiple comparisons across space and with cluster size (*k*) ≥ 30 voxels. Subsequently, in order to further confirm our results, we computed in each subject mean values across voxels of significant comparison clusters and applied Bonferroni correction for multiple comparisons. Age and sex were set as covariates in all the analyses. Brain regions corresponding to local maxima within significant clusters were anatomically mapped using standard-space atlases provided by FSL (JHU DTI-based WM atlases for WM; Harvard–Oxford cortical/subcortical structural atlases for GM).

## Results

### General

No significant differences were found between OHT and NC in terms of age (*p* = 0.85), sex (*p* = 0.9), and grade of WM hyperintensities (*p* = 0.44; grade 0: 61% vs. 72.4%; grade 1: 39.9% vs. 24.1%; grade 2: 0% vs. 3.5%; no grade 3 in either groups) (Table 1).

**TABLE 1:** Comparison of demographic and MRI characteristics between OHT subjects and NC.

	**OHT (*n* = 18)**	**NC (*n* = 29)**	**Stats**
Age, years (mean ± SD)	58.3 ± 9.8	57.9 ± 9.9	*p* = 0.85
Sex (M/F)	10/8	15/14	*p* = 0.9
WM hyperintensities			*p* = 0.44
Grade 0	61%	72.4%	
Grade 1	39.9%	24.1%	
Grade 2	0%	3.5%	
Grade 3	0%	0%	
Anatomical connectivity	No voxelwise differences		*p* > 0.005 uncorr
GM matter volumes	No voxelwise differences		*p* > 0.005 uncorr
**Functional connectivity**			
Short range	OHT<NC in DMN, WMN, VAN, SN		*p* < 0.001
Long range	OHT<NC between DMN and SN OHT>NC between VIS I and II		*p* = 0.0012 *p* = 0.0008

*See text for details and abbreviations.*

### Voxelwise Differences of Anatomical Connectivity

No differences in FA at TBSS analysis across the whole brain were found between OHT and NC (Table 1).

### Voxelwise Differences of GM Volumes

No differences in GM volumes across the whole brain were found between OHT and NC (Table 1).

### Voxelwise Differences of Large-Scale Functional Connectivity

Across the whole study population, 14 functionally relevant RSNs were found, including default mode network (DMN) and sub-networks (*n* = 3), medial frontal executive control network (ECN), salience network (SN), right and left frontoparietal working memory network (WMN), dorsal attention network (DAN), ventral attention network (VAN), visual networks [VN, primary (I) and secondary (II) (*n* = 2)], medial temporal (limbic) network (LN), and cerebellar network (CN).

**FIGURE 1:**
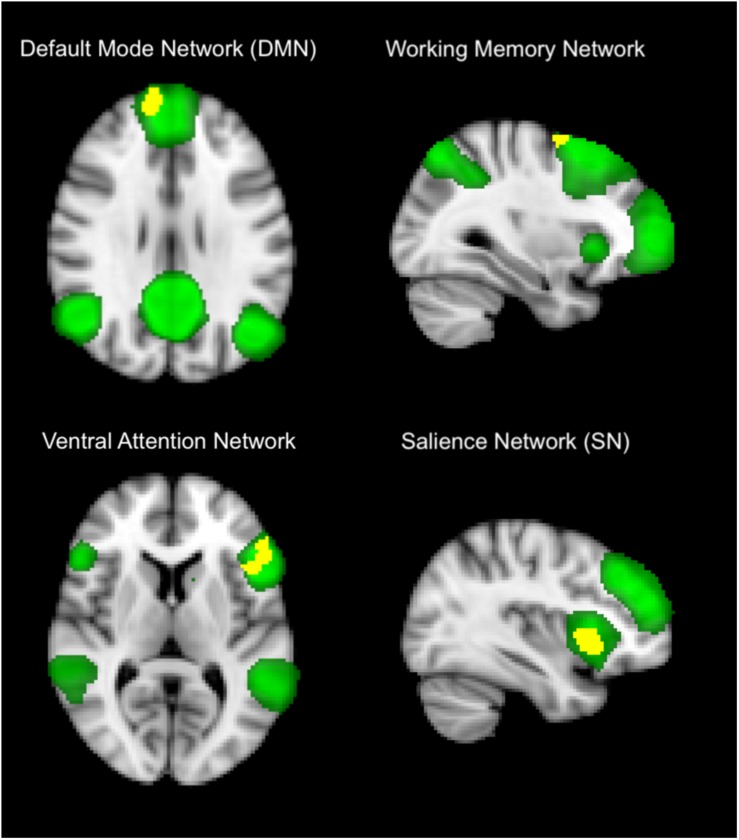
Differences in within-network (short-range) functional connectivity between OHT and NC groups. Yellow shows clusters where the former group has significantly lower functional connectivity than the latter in brain networks (in green), including default mode network **(top left)**, frontoparietal working memory network, **(top right)**, ventral attention network **(low left)**, and salience network **(low right)**. Background image, in radiological convention, is the standard MNI brain. The most informative slices are shown. See section “Results” and Table 1 for more details and text for abbreviations.

Within-network (short-range) functional connectivity of OHT subjects was, compared to NC (Figure 1 and Tables 1, 2), lower (*p* < 0.001 for all) in the DMN (frontal pole, 7.6 ± 1.3 vs. 15 ± 1), frontoparietal WMN (middle frontal gyrus, 3.5 ± 4.3 vs. 9.3 ± 5), VAN (inferior frontal gyrus, frontal pole, and precuneous cortex, 6.3 ± 2.6 vs. 11.7 ± 4), and SN (insular cortex, 2.4 ± 2.4 vs. 6.7 ± 3).

**TABLE 2:** GM regions of RSNs where OHT subjects showed significant lower short-range functional connectivity compared to NC at independent component analysis across the whole brain.

**GM regions (local maxima)**	**MNI *X*,*Y*,*Z* (mm)**	**Side**	**Lobe**	**Cluster size (voxel no.)**	***p* value**
**Default mode network**					
Frontal pole	10,70, −8	R	Frontal	1078	<0.001
**Working memory network**					
Middle frontal gyrus	30,0,62	R	Frontal	62	<0.001
**Ventral attention network**					
Inferior frontal gyrus	−54,32,10	L	Frontal	395	<0.001
Frontal pole	−40,42, −8	L	Frontal	351	<0.001
Precuneous cortex	−6, −62,34	L	Parietal	81	<0.001
**Salience network**					
Insular cortex	−32,14, −8	L	Insula	544	<0.001

*See text for details (including values of functional connectivity) and abbreviations.*

**FIGURE 2:**
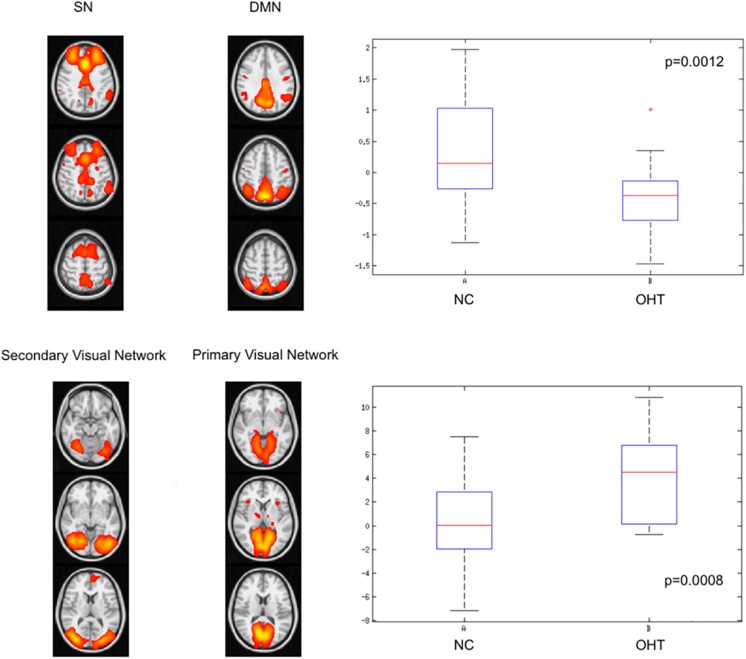
Differences in internetwork (long-range) functional connectivity between OHT and NC groups. The former group has, as shown by the corresponding box-and-whiskers plots of median and range values, lower correlation strength between default mode and salience networks **(upper lower)** and higher correlation between primary and secondary visual networks **(lower panel)**. See text for details and abbreviations.

Internetwork (long-range) functional connectivity of OHT subjects was, compared to NC (Figure 2 and Table 1), lower between DMN and SN [median (range): −0.37 (from −1.46 to 1) vs. 0.14 (from −1.13 to 1.97), *p* = 0.0012] and higher between primary and secondary VN [median (range): 4.5 (from −0.7 to 10.8) vs. 0.02 (from −7.2 to 7.5), *p* = 0.0008].

## Discussion

The traditional view of glaucoma as pure ocular condition with damage to optic nerve head and retinal ganglion cells has been recently challenged and expanded by various advanced MRI studies, which demonstrated the presence of a diffuse neurodegeneration process across the brain, also involving non-visual system (Nuzzi et al., 2018).

In this scenario, OHT may be considered a unique model along the glaucoma spectrum, at a stage where no clinical evidence is present and possibly representing a presymptomatic condition, in view of the fact that a percentage (10%) of subjects with untreated OHT develop POAG within 5 years. Previous findings on glaucoma animal models showed an IOP-sensitive increase in amyloid beta (Ab) (Gupta et al., 2016), whose accrual and spreading is a well-known process in the brain of Alzheimer disease, a typical neurodegenerative brain condition.

To our knowledge, the current study is the first one to explore structural integrity and functional connectivity in the brain of subjects with OHT.

Recent studies in a mouse model of glaucoma reported that actually the first pathogenic process, even before retinal ganglion cell loss, is a synaptopathy (Della Santina et al., 2013), leading to alterations of both structure (degeneration of dendritic arbor and axons of retinal ganglion cells) and function (remodeling of retinal circuitry) (Jakobs et al., 2005).

In our study, OHT subjects did not show, compared to NC, differences across brain in both GM volume and microstructural integrity along WM tracts (anatomical connectivity), as assessed by FA, suggesting that, at this stage along the glaucoma spectrum, raised IOP turns out to be unable and/or insufficient to lead to macro- and microstructural brain damage.

In our previous studies, using the same MRI methodology, we found in both visual and non-visual systems altered anatomical connectivity since mild/early POAG, especially in severe/advanced POAG but also in NTG, both of them also showing atrophy in GM regions key to cognition (e.g., hippocampus, frontal cortex) (Frezzotti et al., 2014, 2016; Giorgio et al., 2018).

Unlike structural damage, in the current study, we otherwise demonstrated, in visual and non-visual networks of the OHT brain, alterations of functional connectivity. In particular, this was decreased in regions of cognition-related networks such as DMN, fronto-parietal WMN, VAN, and SN.

The largest cluster of difference was found at the level of the frontal pole as part of DMN. This is the most studied network of the human brain, which is involved in internal modes of cognition and also activated during internally focused tasks, including memory retrieval. Decreased functional connectivity in DMN was also shown in our previous study on mild/early stage POAG (Frezzotti et al., 2016).

The second largest cluster was found in the insular cortex, part of the so-called SN, which is normally involved in detecting and filtering salient emotional and sensory stimuli.

Other relatively large clusters of decreased functional connectivity in OHT subjects mapped on regions of the VAN (inferior frontal gyrus, frontal pole, and precuneous cortex) and, to a lesser extent, of fronto-parietal WMN (middle frontal gyrus).

The VAN is implicated in visuospatial stimulus-driven attentional control and normally activates upon detection of behaviorally relevant targets, especially when they are salient or unexpected (Fox et al., 2006).

Frontoparietal WMN actually corresponds to the dorsal visual stream, contains a detailed map of the visual field, and is part of the neural processing of vision (“how pathway”) engaged in processing spatial location of objects in order to program behavior.

Decreased functional connectivity in WMN, which was also found in our previous study on mild/early stage POAG (Frezzotti et al., 2016), mapped here in our OHT subjects on the middle frontal gyrus, home to dorsolateral prefrontal cortex, which is mainly involved in general working memory (Potkin et al., 2009) but also in working memory for representation of visual space (Leavitt et al., 2018) and in visual conjunction search (Kalla et al., 2009).

As for long-range functional connectivity, we showed an opposite behavior in two network pairs, with a decrease between default mode and SNs and an increase between primary and secondary VN.

The first finding is in line with decreased short-range functional connectivity shown in SN. In terms of internetwork connection, SN is physiologically involved in modulating the switch between two brain networks, such as DMN and central executive network, which respectively, subserve internally and externally directed cognition.

Overall interpretation of decreased functional connectivity in non-visual networks, at both short range for various networks and at long range between DMN and SN, may be linked to one aspect of neuroplasticity for which compensatory adaptive functional reorganization may not yet be needed in the absence of clinical deficits. In this view, such a process might be only temporarily downregulated, thus reflecting a model of functional reserve, which may be called into action just at a later stage, in case of occurrence of a clinical deficit.

On the other hand, as mentioned above, we also found increased long-range functional connectivity between primary and secondary VN.

Functionally, primary visual network is involved in the early visual processing, by receiving visual information from the lateral geniculate nucleus and sending such information to the secondary visual network.

Unlike decreased functional connectivity explained above, increased functional connectivity between primary and secondary VN may represent another aspect of neuroplasticity, in the form of attempt of adaptive functional reorganization in the visual system at a very early stage, when clinical deficits have not occurred yet.

The main limitation of this study lies in the small sample size. Indeed, it is worth stressing here that this is a pilot study with an “exploratory” nature and findings need to be confirmed in larger cohorts. Moreover, the clinical relevance of these MR findings can be fully appreciated only with a longitudinal observation so that more pronounced MRI abnormalities may be predictive of a future progression to glaucoma. However, we should consider that even a long-term longitudinal study might not represent the solution to this issue as the progression to glaucoma may occur after a very long time or even not be appreciated during lifetime (McMonnies, 2017).

In conclusion, OHT represents a unique model along the glaucoma spectrum for gauging the relationship between brain structural damage, visual function, and neuroplasticity. Our findings suggest the occurrence in the brain of these “asymptomatic” subjects of altered short- and long-range functional connectivity in visual and non-visual networks. Assessing temporal dynamics of brain connectivity changes in OHT subjects with or without conversion to POAG through longitudinal studies will certainly help disentangle the aforementioned relationship. Finally, the absence of brain structural damage in these subjects may represent a window of opportunity for therapeutic intervention in order to interfere with the mechanisms of neurodegeneration at a very early stage of glaucoma.

## Data Availability Statement

The raw data supporting the conclusions of this article will be made available by the authors, without undue reservation, to any qualified researcher.

## Ethics Statement

The studies involving human participants were reviewed and approved by the Ethics Committee of the Azienda Ospedaliera Universitaria Senese. The patients/participants provided their written informed consent to participate in this study.

## Author Contributions

AG, ND, and PF: study concept and design. AG, JZ, FC, and PF: data acquisition and analysis. AG and ND: drafting the manuscript. AG, JZ, FC, ND, and PF: critical revision and approval of the final manuscript.

## Conflict of Interest

The authors declare that the research was conducted in the absence of any commercial or financial relationships that could be construed as a potential conflict of interest.

## Footnotes

1 www.nitrc.org/projects/dtiprep/

2 www.fmrib.ox.ac.uk/fsl/

3 https://github.com/rhr-pruim/ICA-AROMA

4 http://fsl.fmrib.ox.ac.uk/fsl/fslwiki/MELODIC

5 http://fsl.fmrib.ox.ac.uk/fsl/fslwiki/FSLNets
